# 1304. Early Surgical Procedures and Clinical Outcomes Among Children Hospitalized with Acute Hematogenous Osteomyelitis

**DOI:** 10.1093/ofid/ofad500.1143

**Published:** 2023-11-27

**Authors:** Justin B Searns, Matt Hall, Meghan C Birkholz, Brittany Hubbell, Andrew S Kern-Goldberger, Matthew Kronman, Jessica Markham, Stephanie L Rolsma, Samir S Shah, Marie E Wang, Sean O’Leary, Samuel R Dominguez, Sarah K Parker

**Affiliations:** Children’s Hospital Colorado, University of Colorado, Aurora, Colorado; Children's Hospital Association, Lenexa, Kansas; Children's Hospital Colorado, Clifton, Virginia; Cincinnati Children's Hospital Medical Center, Mason, Ohio; Cleveland Clinic Children's Hospital, Cleveland, Ohio; Seattle Children's Hospital / University of Washington, Seattle, Washington; Children's Mercy Kansas City, Kansas City, Missouri; Vanderbilt University Medical Center, Nashville, Tennessee; Cincinnati Children's Hospital Medical Center, Mason, Ohio; Stanford University School of Medicine, Palo Alto, California; University of Colorado School of Medicine, Aurora, Colorado; University of Colorado/Children's Hospital Colorado, Aurora, CO

## Abstract

**Background:**

Surgical biopsy and drainage may have diagnostic and therapeutic benefits for children with acute hematogenous osteomyelitis (AHO). However, it remains unknown whether routinely expediting surgical procedures improves clinical outcomes compared to a “wait-and-see” approach. We aimed to determine whether hospitals that frequently perform early surgery for AHO have improved clinical outcomes compared to hospitals that rarely perform early surgery.

**Methods:**

Admissions for AHO at 48 hospitals were identified in the Pediatric Health Information System from 1/2015 through 10/2022 for patients 6 months to 18 years with a discharge diagnosis of osteomyelitis using a previously validated algorithm to exclude chronic osteomyelitis, non-hematogenous infections, and significant comorbidities. Encounters assigned a surgical procedure code on hospital day 0 or 1 were classified as undergoing “early surgery” and hospitals were stratified based on proportion of AHO patients receiving early surgery. Clinical outcomes at the 16 hospitals with the lowest rates of early surgery (rare group) were compared to those from the 16 hospitals with the highest rates of early surgery (frequent group). Categorical variables were compared using chi-square tests and continuous with Wilcoxon Rank-Sum tests.

**Results:**

There were 2102 encounters in the rare group and 2040 encounters in the frequent group. Median proportion of AHO patients undergoing early surgery was 20% in the rare group compared to 37% in the frequent group (Range 10% to 54%). Length of stay in the frequent group was marginally lower (4.4 vs 4.1 days, P < 0.001), as was length of intravenous antimicrobials (4.5 vs 4.3 days, P< 0.001). Patients were more likely to be critically ill at frequent early surgery hospitals (1% vs 5%, P< 0.001), more likely to have a central line placed (8% vs 11%, P=0.002), and more likely to have a pathogen identified (50% vs 59%, P< 0.001). Cost per admission was slightly higher in the frequent group ($12960 vs $13759, P< 0.001).

**Figure:**
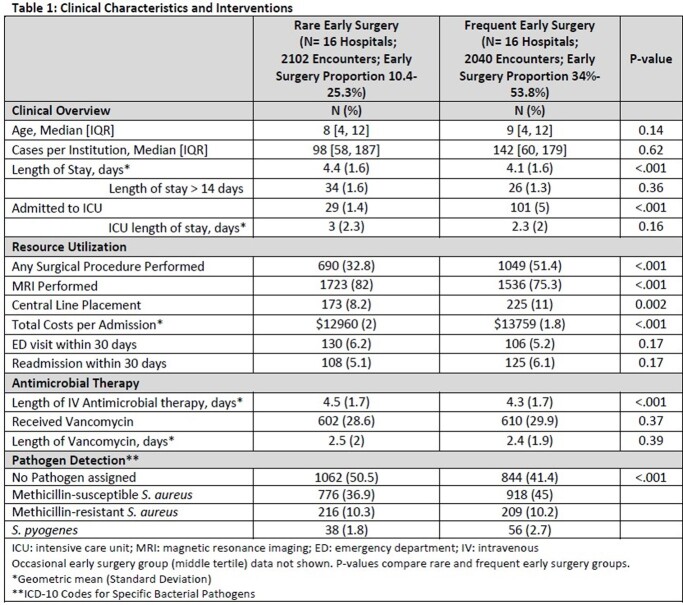

**Conclusion:**

Frequency of early surgical procedures for pediatric AHO varies across institutions. Clinical outcomes and resource utilization for children with AHO are similar between institutions that rarely and frequently expedite surgical procedures.

**Disclosures:**

**Samuel R. Dominguez, MD, PhD**, Biofire Diagnostics: Advisor/Consultant|Biofire Diagnostics: Grant/Research Support|Cobio Diagnostics: Board Member|Karius: Advisor/Consultant|Pfizer: Grant/Research Support

